# Trends in Suicidal Mortality and Motives among Working-Ages Individuals in Japan during 2007–2022

**DOI:** 10.3390/ejihpe13120193

**Published:** 2023-11-27

**Authors:** Ryusuke Matsumoto, Eishi Motomura, Toshiaki Onitsuka, Motohiro Okada

**Affiliations:** 1 Department of Neuropsychiatry, Division of Neuroscience, Graduate School of Medicine, Mie University, Tsu 514-8507, Japan; matsumoto-r@clin.medic.mie-u.ac.jp (R.M.); motomura@clin.medic.mie-u.ac.jp (E.M.); 2 Department of Psychiatry, NHO Sakakibara National Hospital, Tsu 514-1292, Japan; onitsuka.toshiaki.vc@mail.hosp.go.jp

**Keywords:** suicide, COVID-19, Japan, working-age, child-raising

## Abstract

Suicides in Japan consistently decreased from 2009–2019, but increased during the COVID-19 pandemic. To identify causes of increasing suicides, age-dependent and temporal fluctuations of suicide mortality rate per 100,000 (SMRP) in working-age generations (20–59 years) disaggregated by suicidal motives (7-categories; 52-subcategories) and sex from 2007 to 2022, were analyzed by analysis of variance and joinpoint regression, respectively, using the government suicide database “Suicide Statistics”. The SMRP of 20–29 year-old males and 20–49 year-old females began to increase in the late 2010s. SMRPs of these high-risk groups for suicides caused by depression (the leading suicidal motive for all groups) began increasing in the late 2010s. Economic-related, employment-related, and romance-related problems contributed to the increasing SMRPs in 20–29 males in the late 2010s. Romance-related and family-related problems contributed to the increasing SMRPs of 20–29 females in the late 2010s. Increasing SMRPs caused by child-raising stress in 20–39 year-old females from the late 2010s was a remarkable finding. In contrast, SMRPs of 30–59 year-old males consistently decreased until 2021; however, in these groups, SMRPs for suicides caused by various motives sharply increased in 2022. The consistent increase in SMRPs of high-risk groups from the late 2010s to the pandemic suggest recent socioeconomic and psychosocial problems in Japan possibly contributed to the increasing SMRPs in these high-risk groups independently of pandemic-associated factors, whereas the SMRPs of males of 30–59 years were probably associated with the ending of the pandemic rather than pandemic-associated factors.

## 1. Introduction

Following the asset bubble collapse (1991), Great Hanshin earthquake (1995), and Asian economic crisis (1997), annual suicides in Japan drastically increased from 24,391 (1997) to 32,863 (1998) [1,2,3,4,5,6]. Until 2009, the annual number of suicides exceeded 30,000 in Japan [7,8,9]. In this background, the Japanese government enacted the ‘Basic Act on Suicide Prevention’ (2006) and ‘General Policies for Comprehensive Measures against Suicide’ (2007) [1], and the Ministry of Health, Labor and Welfare (MHLW) started to contribute funds to prefectures/municipalities to enhance regional suicide prevention programs in the form of ‘Emergency Fund to Enhance Community-Based Suicide Countermeasure’ [1,6,10,11,12,13,14]. Since 2009, annual suicides have consistently decreased to 20,169 (2019) [2,3,4,5,15]. However, they increased after the COVID-19 pandemic outbreak [16,17,18,19,20,21,22,23,24,25,26,27,28,29]. In the initial stage of the pandemic (2020–2021), although annual male suicides continuously decreased from 14,055 (2019) to 13,939 (2021), female suicides increased from 6091 (2019) to 7068 (2021). Conversely, in 2022, the annual suicides of both males (14,746) and females (7135) increased [1,22,30]. Recently, various statistical analyses have reported that the younger generation (<30 years) [31] and females of 30–49 years were at risk for suicide during the pandemic [16,17,18,19,20,21,22]. These high-risk groups historically have relatively fewer suicide mortalities in comparison to working-age males, and the associated psychosocial/socioeconomic factors remain to be clarified [32,33,34,35,36,37].

Our recent analysis revealed fluctuations in the suicide mortality rate per 100,000 (SMRP) in the young generation showed at least three temporal patterns, the onset of increasing suicide before the pandemic, synchronized with the pandemic outbreak (2020), and in the late phase of the pandemic (2022) [18,20,21,22,23,38,39]. Therefore, although national-level suicides in Japan increased approximately six months after the pandemic outbreak (October 2020) [18,19,21], it cannot be definitively concluded that the pandemic is a primary impactable factor associated with increasing suicide in high-risk groups [20,22,23]. Valid analyses of the causality of these increases in high-risk groups could contribute to evidence-based suicide prevention programs [20,21,22,40,41,42]. Therefore, to identify factors associated with the recent increasing suicides in Japan, we analyzed a government suicide database to determine the temporal fluctuations in the SMRPs of working-age generations caused by suicidal motives disaggregated by age/sex in 2007–2022.

## 2. Materials and Methods

This cross-sectional study adhered to the Strengthening the Reporting of Observational Studies in Epidemiology (STROBE) Reporting Guidelines. The Medical Ethics Review Committee of Mie University waived the requirement for informed consent and ethical approval because the government data are publicly available.

### 2.1. Database

Annual suicide numbers disaggregated by motive, sex (males and females), and age (20–29, 30–39, 40–49, and 50–59 years) in Japan between 2007 and 2022 were obtained from the ‘Suicide Statistics’ (SSNPA) database, which is collected by the National Police Agency [7,30,39,43]. The SSNPA provides annual suicide numbers disaggregated by suicidal motives (seven categories: family-related, health-related, economic-related, employment-related, romance-related, school-related, and other motives, with 52 subcategories) (Supplementary Tables S1–S4) [1,7,11,30,39,43].

The police must investigate the personal characteristics and background factors of each suicide case. Since it is impossible to collect suicide motives from the victims themselves, to eliminate subjectivity as much as possible, the police investigate suicide motives based on evidence, suicide notes, official documentation (e.g., medical certificates and clinical recordings) and testimony from the victim’s family [7,39,43,44]. The results of this investigation discuss the different motives for suicide, and these motives are compared to previously compiled lists of motives for suicide (52 subcategories in SSNPA). The majority of suicides have diverse causes, backgrounds and complications, and occur with interactions among various factors. Therefore, the SSNPA is permitted to count multiple probable causes/motives per a suicide. The SSNPA published the annual suicide numbers disaggregated by 52 suicidal motives, sex (males and females) and age (20–29, 30–39, 40–49, and 50–59 years old) during 2007–2022. Detailed explanations of the suicide motives have been described in previous reports [11,39,43], and detailed lists of categories and standard suicide rates per 100,000 population disaggregated by motive, sex, and age are described in Supplementary Tables S1–S4.

The populations in Japan disaggregated by sex (males and females) and age (20–29, 30–39, 40–49, and 50–59 years old) during 2007–2022 were obtained from the “Surveys of Population, Population Change and the Number of Households based on the Basic Resident Registration” of the System of Social and Demographic Statistics generated by the Statistics Bureau of the Ministry of Internal Affairs and Communications (SBMIAC) [45]. The average annual incomes of permanent and non-permanent employees, disaggregated by age and sex, were obtained from the ‘Basic Survey on Wage Structure’ of the MHLW [46]. Employment rates of working-age males and females were obtained from the ‘White Paper on Gender Equality 2023’ [47,48,49] published by the Cabinet Office. Annual employment rates of males and females during 2007–2022 were obtained from the “White Paper on Gender Equality 2023” published by the Cabinet Office [47,48,49,50].

### 2.2. Statistical Analysis

The suicide mortality rate per 100,000 (SMRP) was calculated by dividing the annual suicide numbers of the target group by the annual populations of the same target group in the same year. SSNPA released annual suicide numbers for which motives were determined [20,22].

The trends, discontinuity, and their effect size of SMRPs and complete employment rates (CURs) from 2007 to 2022 were analyzed by joinpoint regression analysis (JPRA) using Joinpoint Regression Program v5.0.2 (National Cancer Institute, Bethesda, MD, USA) [19,22,51,52,53,54]. JPRA fits the simplest joinpoint model that the trend data allows and identifies significant points where trends change. This is a powerful statistical method for detection of unknown joinpoints (transformed trends and discontinuities) [52]. A comprehensive review of the statistics and underlying methodology applied in JPRA have been explained in a review report [52]. The detailed description of the methods used in the Joinpoint Regression Software is described in the user manual published by the National Cancer Institute (NCI) [51]. In this background, the present study analyzed the fluctuations in SMRP during the 2007–2022 using JPRA. A *p* value of <0.05 (two-tailed) was considered to indicate statistical significance.

The differences in SMRPs from 2007 to 2022 were compared using two-way analysis of variance (ANOVA) with Scheffe’s post-hoc-test by SPSS for Windows version 27 (IBM, Armonk, NY, USA) [19,21]. When the F value was significant (*p* < 0.05), the F value of ANOVA was analyzed using Scheffe’s post-hoc analysis [19,21]. A *p* value of <0.05 (two-tailed) was considered to indicate statistical significance.

## 3. Results

### 3.1. SMRPs Disaggregated by Se, Age and Suicidal Motives from 2007–2022

**Figure 1:**
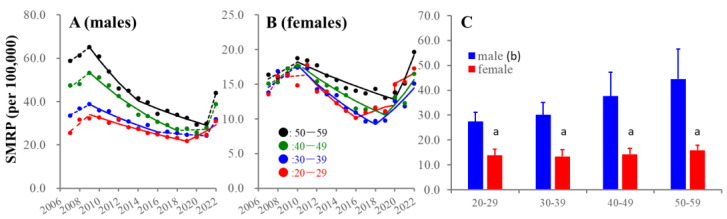
Average and fluctuations of SMRPs of working-age generation from 2007–2022. Temporal fluctuations of SMRPs in working-age generation of males (**A**) and females (**B**) and average of SMRPs (**C**) from 2007 to 2022 in Japan. Ordinates indicate the SMRPs (per 100,000 population). In panels (**A**) and (**B**), abscissas indicate years. Red, blue, green, and black circles indicate the observed annual SMRPs of 20–29, 30–39, 40–49, and 50–59, respectively. Solid and dotted lines indicate the significant (*p* < 0.05) and non-statistically significant trends of SMRPs detected by joinpoint regression analysis (JPRA), respectively. In panel (**C**), a: *p* < 0.05 relative to same age range males, b: *p* < 0.05 statistically significant age-dependency, using two-way analysis of variance (ANOVA) with Scheffe’s post-hoc test.

The SMRPs of all age groups consistently decreased in the early 2010s. Joinpoints of female SMRPs (from decreasing to increasing) of 20–29, 30–39, 40–49, and 50–59 years were detected in 2016, 2018, 2019, and 2020, respectively (Figure 1). In particular, the SMRP for 20–29 females displayed positive discontinuation synchronized with the COVID-19 outbreak. Accordingly, among females, 20–29 females showed the largest SMRP in 2020–2021. Joinpoints of male SMRPs (from decreasing to non-statistically significant changes) for 30–39 and 40–49 years were detected in 2016 and 2018, respectively (Figure 1). These groups displayed sharply (drastic but non-significant) increasing in 2022. Similarly, the SMRP of 50–59 males consistently decreased until 2021, but sharply increased in 2022 (Figure 1). The joinpoint of SMRPs (from decreasing to increasing) of 20–29 males was detected in 2019 (Figure 1).

### 3.2. Age- and Sex-Dependent Impacts of Suicidal Motives

**Figure 2:**
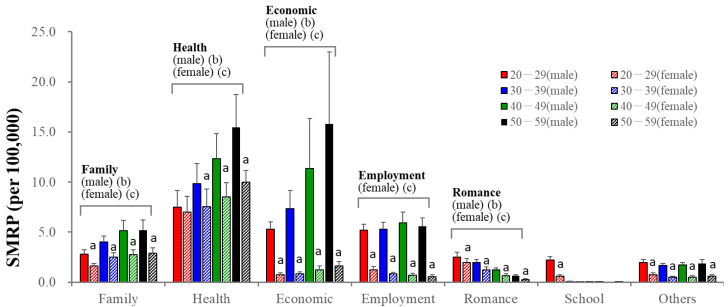
SMRPs caused by major 7 categorized motives of males (filled columns) and females (striped columns). Red, blue, green and black columns indicate the average of SMRPs from 2007 to2022 of 20–29, 30–39, 40–49, and 50–59 populations, respectively. Ordinate indicates mean ± SD of SMRPs from 2007 to 2022. a: *p* < 0.05 relative to males of same age group, b: *p* < 0.05 statistically significant age-dependency in males, c: *p* < 0.05 statistically significant age-dependency in females detected by two-way ANOVA with Scheffe’s post-hoc test.

Among categorized motives, health-related problems were the leading suicidal motive of all groups (Figure 2). Economic-related and family-related problems were the second leading motives for males and females of 30–59 years, respectively, whereas romance-related problems were the second leading motive for 20–29 females (Figure 2). SMRPs caused by health-related motives at 20–29 years were almost equal in males and females, whereas other SMRPs of males were larger than those of females (Figure 2). SMRPs caused by family-, health- and economic-related motives increased in an age-dependent manner in both sexes; however, SMRPs caused by romance-related motives decreased in an age-dependent manner in both sexes (Figure 2). Female SMRPs caused by employment-related motives also decreased in an age-dependent manner, whereas those of males did not change (Figure 2).

**Figure 3:**
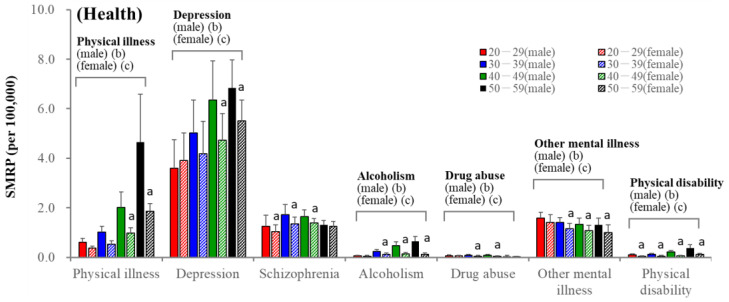
SMRPs caused by subcategorized health-related motives of males (filled columns) and females (striped columns). Red, blue, green, and black columns indicate the mean ± SD of SMRPs from 2007 to 2022 of 20–29, 30–39, 40–49, and 50–59 populations, respectively. Ordinate indicates mean ± SD of SMRPs from 2007 to 2022. a: *p* < 0.05 relative to males of same age group, b: *p* < 0.05 statistically significant age-dependency in males, c: *p* < 0.05 statistically significant age-dependency in females detected by two-way ANOVA with Scheffe’s post-hoc test.

Among subcategorised motives, depression was the leading impact factor in all groups. SMRP caused by depression was slightly higher in males, although depression contributed 30% of the total female SMRPs (vs. 15% in males) (Figure 3). Other mental illness and physical illness were the second leading motive for individuals of 20–29 and 50–59 years, respectively, in both sexes (Figure 3). The impact of depression and physical illness increased in an age-dependent manner. SMRPs for individuals of 20–29 and 30–39 years caused by depression and physical illness were almost equal in males and females, whereas these rates in 40–59 males were higher than those in females (Figure 3). Conversely, SMRPs caused by other mental illnesses decreased in an age-dependent manner and were almost equal in males and females of 20–29 years (Figure 3). 

**Figure 4:**
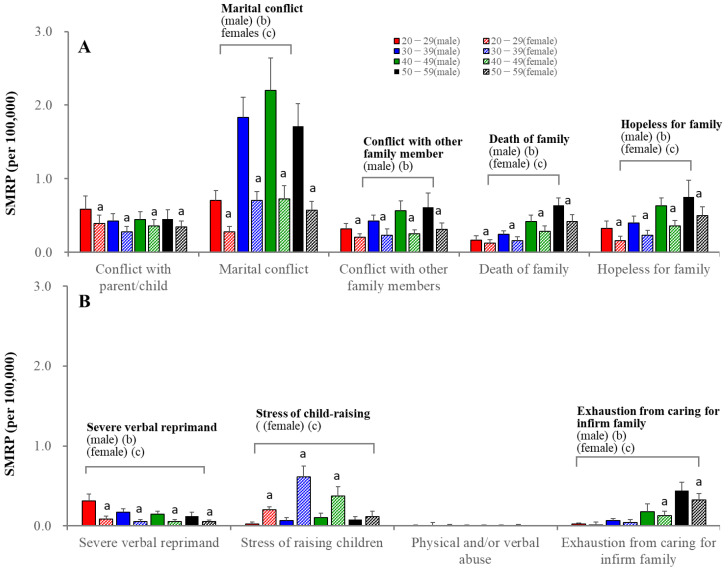
SMRPs caused by subcategorized family-related motives of males (filled columns) and females (striped columns). Red, blue, green, and black columns indicate the mean ± SD of SMRPs from 2007 to 2022 of 20–29, 30–39, 40–49, and 50–59 populations, respectively. Ordinates indicate mean ± SD of SMRPs from 2007 to 2022. a: *p* < 0.05 relative to males of same age group, b: *p* < 0.05 statistically significant age-dependency in males, c: *p* < 0.05 statistically significant age-dependency in females detected by two-way ANOVA with Scheffe’s post-hoc test. Panel (**A**) indicates the SMRPs caused by conflict with parent/child, maternal conflict, conflict with other family members, death of family and hopeless for family. Panel (**B**) indicates SMRPs caused by severe verbal reprimand, stress of raising children, physical and/or verbal abuse and exhaustion from caring for infirm family.

Among subcategories in family-related motives, marital conflict was an impactable motive, followed by child-raising stress in 20–49 females (Figure 4). The SMRP for marital conflict peaked at 40–49 years in both sexes, whereas the SMRP for child-raising stress peaked at 30–39 years in females (Figure 4). 

**Figure 5:**
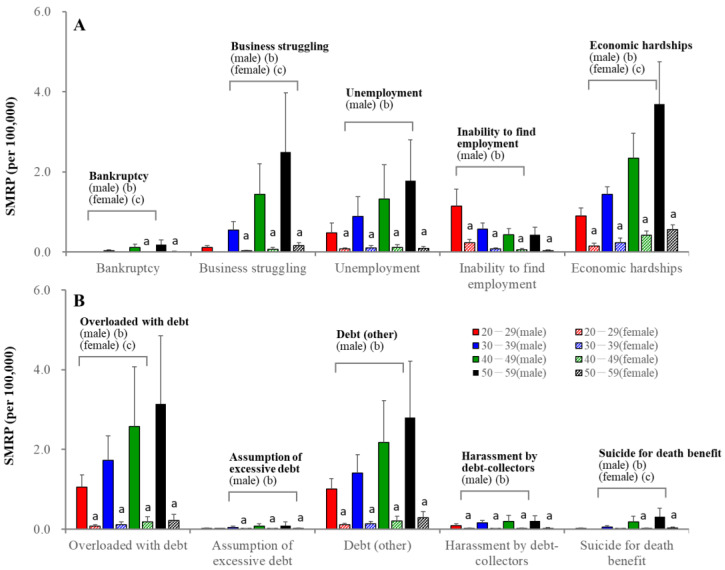
SMRPs caused by subcategorized economy-related motives of males (filled columns) and females (striped columns). Red, blue, green, and black columns indicate the mean ± SD of SMRPs from 2007 to 2022 of 20–29, 30–39, 40–49, and 50–59 populations, respectively. Ordinates indicate mean ± SD of SMRPs from 2007 to2022. a: *p* < 0.05 relative to males of same age group, b: *p* < 0.05 statistically significant age-dependency in males, c: *p* < 0.05 statistically significant age-dependency in females detected by two-way ANOVA with Scheffe’s post-hoc test. Panel (**A**) indicates the SMRPs caused by bankruptcy, business struggling, unemployment, inability to find employment and economic hardship. Panel (**B**) indicates SMRPs caused by overloaded with debt, assumption of excessive debt, debt (other), harassment by debt-collectors and suicide for death benefit.

Economic problems were impactable motives for males but were limited for females (Figure 5). Among economic-related motives, economic hardship was the leading impactable motive, followed by debt overload in males of 30–39 years. SMRPs caused by these factors increased in an age-dependent manner. However, in 20–29 males, inability to find employment was the leading motive, followed by overload with debt and economic hardship (Figure 5). SMRPs caused by the inability to find employment decreased in an age-dependent manner in both sexes (Figure 5).

### 3.3. Fluctuations in SMRPs Caused by Health-Related Motives

**Figure 6:**
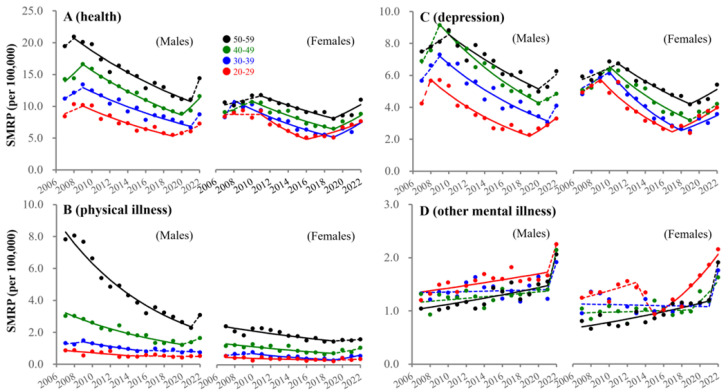
Fluctuations of SMRP caused by health-related motives from 2007–2022. Fluctuations of SMRPs caused by health-related motive (**A**), physical illness (**B**), depression (**C**) and other mental illness (**D**) of working-age males and females. Red, blue, green, and black circles indicate the observed annual SMRPs of 20–29, 30–39, 40–49, and 50–59 populations, respectively. Solid and dotted lines indicate the significant (*p* < 0.05) and non-statistically significant trends of SMRPs between 2007–2022 detected by JPRA, respectively. Ordinates indicate the SMRP (per 100,000 population). Abscissas indicate years.

The joinpoint of SMRPs caused by health-related motives in females of 30–59 years was detected in 2019, whereas it was observed at 20–29 years before the pandemic. The joinpoints of male SMRPs of 30–59 years caused by health-related motives were also observed after the pandemic outbreak, but that of 20–29 years males was in 2019 (Figure 6). The joinpoints of SMRPs caused by depression in 30–59 years males were observed after the pandemic outbreak, whereas they were observed at 20–29 years in males and females in 2019 and 2016, respectively (Figure 6). SMRPs caused by health-related motives and depression in females of 20–29 years were the lowest until the early 2010s, but were larger than those in 30–39 year-old females during the pandemic. In the late 2010s, SMRPs caused by other mental illness increased at 20–29 and 50–59 years, but indicated no significant change at 30–49 years (Figure 6). During the pandemic, SMRPs caused by other mental illnesses increased sharply in all groups (Figure 6).

### 3.4. Fluctuations in SMRPs Caused by Family-Related Motive

**Figure 7:**
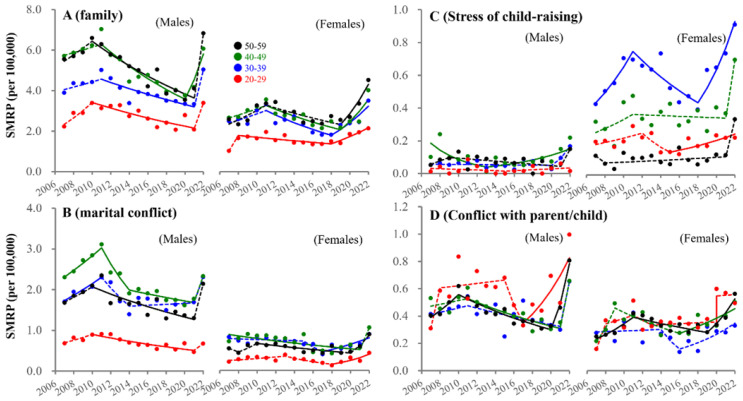
Fluctuations of SMRP caused by family-related motives between 2007–2022. Fluctuations of SMRPs caused by family-related motive (**A**), marital conflict (**B**), child-raising stress (**C**) and conflict with parent/child (**D**) of working-age males and females. Red, blue, green, and black circles indicate the observed annual SMRPs of 20–29, 30–39, 40–49, and 50–59 populations, respectively. Solid and dotted lines indicate the significant (*p* < 0.05) and non-statistically significant trends of SMRPs from 2007 to 2022 detected by JPRA, respectively. Ordinates indicate the SMRP (per 100,000 population). Abscissas indicate years.

Male SMRPs caused by family-related subcategories sharply increased after the pandemic outbreak, whereas exceptionally, suicide caused by conflict with parent/child in males of 20–29 years began increasing in 2017 (Figure 7). In contrast, female SMRPs caused by family-related motives began increasing before the pandemic. In particular, SMRPs caused by child-raising stress in females of 20–29 and 30–39 years began increasing in 2015 and 2018, respectively, whereas females of 40–59 years showed a sharp increase in 2022 (Figure 7). SMRPs caused by marital conflict in females of 20–39 years also began increasing before the pandemic, whereas females of 40–59 showed a sharp increase during the pandemic (Figure 7). SMRPs caused by conflict with parent/child began increasing in 20–29 year-old males in 2017. In contrast, females showed positive discontinuity synchronized with the pandemic outbreak (Figure 7). 

### 3.5. Fluctuations in SMRPs Caused by Economy-Related Motive

**Figure 8:**
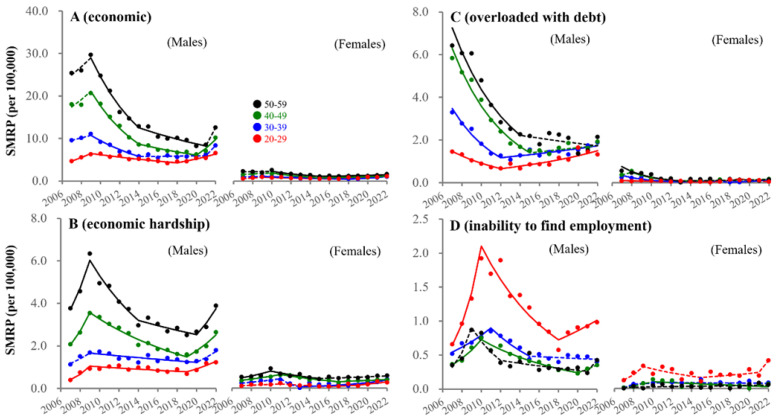
Fluctuations of SMRP caused by economic-related motives from 2007–2022. Fluctuations of SMRPs caused by economic-related motive (**A**), economic hardship (**B**), overloaded with debt (**C**) and inability to find employment (**D**) of working-age males and females. Red, blue, green, and black circles indicate the observed annual SMRPs of 20–29, 30–39, 40–49, and 50–59 populations, respectively. Solid and dotted lines indicate the significant (*p* < 0.05) and non-statistically significant trends of SMRPs from 2007 to 2022 detected by JPRA, respectively. Ordinates indicate the SMRP (per 100,000 population). Abscissas indicate years.

**Figure 9:**
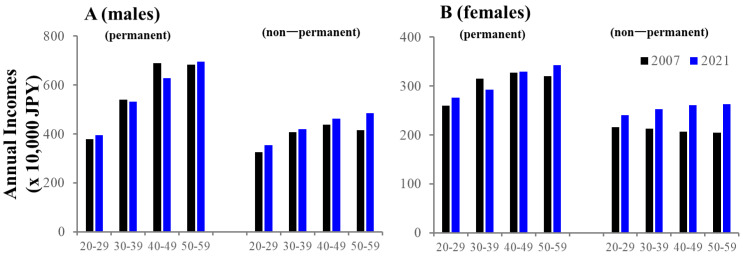
Comparison of annual incomes of males (**A**) and females (**B**) between 2007 and 2021. Average of annual incomes in 2007 (black) and 2021 (blue). Ordinates indicate the incomes (x 10,000 JPY). Panels (**A**) and (**B**) indicate males and females, respectively.

In males of 20–29 years, SMRPs caused by economic-related motives began increasing in 2018, whereas males of 30–59 years showed a sharp increase in 2022. SMRPs caused by economic hardship consistently decreased in all male groups before the pandemic, but increased with the pandemic (Figure 8). In particular, SMRPs caused by inability to find employment and overload with debt in 20–39 year-old males began to increase before the pandemic (Figure 8). In females, SMRPs caused by economic-related motives began increasing before the pandemic in all age groups (Figure 8). SMRPs caused by economic hardship began increasing before the pandemic in 20–49 year-old females (Figure 8). However, the annual incomes of permanent and non-permanent workers were almost equal between 2007 and 2021 (Figure 9). 

### 3.6. Fluctuations in SMRPs Caused by Employment-Related and Romance-Related Motives

**Figure 10:**
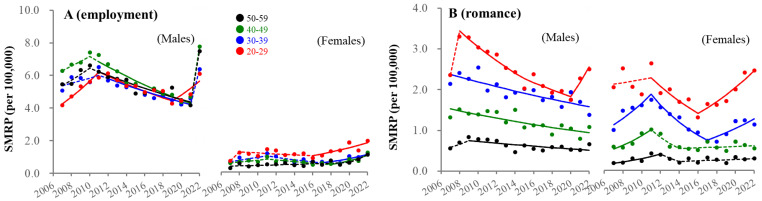
Fluctuations of SMRP caused by employment-related and romance-related motives from 2007–2022. Fluctuations of SMRPs caused by employment-related motive (**A**) and romance-related (**B**) motive of working-age males and females. Red, blue, green, and black circles indicate the observed annual SMRPs of 20–29, 30–39, 40–49, and 50–59 populations, respectively. Solid and dotted lines indicate the significant (*p* < 0.05) and non-statistically significant trends of SMRPs from 2007 to 2022 detected by joinpoint regression analysis (JPRA), respectively. Ordinates indicate the SMRP (per 100,000 population). Abscissas indicate years.

**Figure 11:**
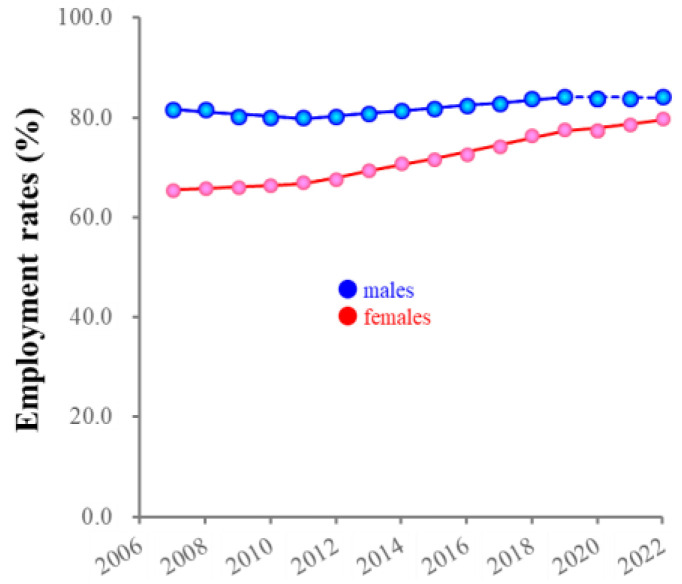
Fluctuations of employment rates for males and females from 2007 to 2022. Red and blue circles indicate the observed annual employment rate of females and males, respectively. Solid and dotted lines indicate the significant (*p* < 0.05) and non-statistically significant trends of employment rates from 2007 to 2022 detected by joinpoint regression analysis, respectively. Ordinates indicate the employment rate (%). Abscissas indicate years.

SMRPs caused by employment-related motives in individuals of 20–29 years began increasing before the pandemic in both sexes (males/females: 2018/2016), whereas 30–59 males showed an increase in 2022 (Figure 10). Employment rates of working-age males increased between 2007–2019, but did not change between 2019 and 2022, whereas those of females consistently increased between 2007 and 2022, resulting in these being almost equal during the pandemic (Figure 11). Romance-related motives decreased in an age-dependent manner in the seven major suicidal motives (heartbreak was the leading motive). In 20–29 year-old males, SMRPs caused by romance-related motive began increasing in 2020, whereas a consistent decrease was observed in males of 30–59 years (Figure 10). In contrast, SMRPs caused by heartbreak began increasing in 20–39 year-old females in 2017 (Figure 10). 

## 4. Discussion

This study identified several contributing suicidal motives for increasing suicides in high-risk groups (males of 20–29 years and females of 20–49 years) during the COVID-19 pandemic in Japan. First, SMRPs of high-risk groups began increasing in the late 2010s, whereas SMRPs of males of 30–59 years did not increase until the early stage of the pandemic, but sharply (non-significantly but drastically) increased in 2022. Increasing trends of SMRPs were pronounced at 20–29 years in both sexes in comparison to other ages, in the early stage of the pandemic (from 2020–2021), females showed their highest SMRP at 20–29 years, while the SMRP of males of 20–29 years was almost equal to that of males of 30–49 years. Second, the leading motive of all groups was internalization symptoms/disorders (mainly depression). SMRPs caused by depression in males of 20–29 years and all female groups began increasing in the late 2010s. The SMRP caused by depression was slightly higher in males, but SMRPs caused by depression contributed 30% of the total female SMRPs and 15% of total male SMRPs. Third, the second leading suicidal motive of females was family-related motives, which began to increase in the late 2010s. Notably, among family-related motives, child-raising stress was an impactable motive for females of 20–49 years, and the SMRPs of 20–39 females caused by child-raising stress began increasing in the late 2010s. Fourth, in individuals of 20–29 years economic- and romance-related problems were the second leading motives in males and females, respectively. They also began increasing before the pandemic outbreak. These results suggest the possibility that recent (from before the pandemic outbreak) socioeconomic/psychosocial problems in Japan may play important roles in increasing SMRPs in males of 20–29 years and females of 20–49 years (high-risk groups during the pandemic), whereas, exceptionally, SMRP of 20–29 years females might be increased by pre-pandemic factors added to pandemic-associated factors during the pandemic. In contrast, the SMRPs of males of 30–59 years were probably affected by factors associated with the ending of the pandemic rather than pandemic-associated factors.

Regarding the increasing SMRPs of females of 20–49 years, work–life balance possibly plays important roles in increasing suicide in these groups. The shared suicidal motives for associated with the increase in SMRPs in females of 20–39 years before the pandemic were depression, child-raising stress, and employment-related motives. In other words, the situation in which role models for housework, child-raising, and social advancement for females have not been established in Japanese society might manifest itself as increasing suicides among at-risk individuals in the child-raising generation. The Japanese gender gap index did not improve from 0.645 (2006) to 0.684 (2023) [47,55]; however, the employment rate of females in the child-raising generation (25–44 years) increased from 65% in 2007 to approximately 80% in 2022, which was almost equal to the rate of working-age males (85%). Improving gender inequality is considered to be achieved by establishing a societally valued “traditionally masculine” roles and liberating them from societally devalued “traditional feminine” roles such as “housework” [56,57,58,59,60]. However, in Japan, it is well known supporting social capital for child-raising has been vulnerable for mothers who wish to prioritize parenting or aspire to balance child-raising and employment in Japan [57,61,62]. Therefore, when parents engage in work that is incompatible with childcare, alternate arrangements for care of their children must be made, leading to increased stress among mothers [63,64]. Additionally, some mothers are unwilling to leave childcare, since childcare represents an investment in the future, and relationships with children are irreplaceable and lifelong [56,65]. Based on the relevant literature, females in Japan participate in the workforce, but from the perspective of work–life balance, they place much higher priority on childcare than employment. Indeed, although the employment rates of males and females were almost equal, the impacts of employment-related motives on females SMRPs were limited compared to those of males.

Internalising symptoms/disorders were reported as major causes of increasing student suicides in the late 2010, in Japan [23,66]. Internalising symptoms/disorders are established suicidal risks for adolescents and young generations [23,33,67,68,69], and their prevalence has been increasing in Japan [23,66]. Therefore, the recent increase in suicides among the young generation involves similar causalities to the increase among adolescents worldwide [33]. Increases in SMRPs caused by other mental illnesses could not be detected in males of 30–49 years or females. Considering the age of onset of internalising disorders was approximately 15 years [70,71,72], this discrepancy between 30–49 years and <30 years suggested that increasing suicides caused by internalising symptoms/disorders in the younger generation may have emerged in the last decade. The increasing SMRPs of females of 20–29 years caused by romance-related and employment-related motives can be interpreted as impairments of inter-relationships via diminished self-concept in internalising symptoms [73]. This assumption may also apply to the increase in SMRPs of males of 20–29 years. Internalising symptoms/disorders and socioeconomic/psychosocial impairments are known to enhance each other [74,75,76]. Although the employment rate and incomes did not decrease at 20–29 years, the increasing SMRPs of males of 20–29 years caused by inability to find employment, economic hardship, overload with debt, and heartbreak were also explained by internalized feelings of personal responsibility for socioeconomic/psychosocial failure [77].

The sharp increase in SMRPs in 2022 requires further consideration. Several studies comparing mental issues before and after the lockdown to explore the long-term consequences of the pandemic reported interesting and comparable findings [78,79,80]. The mental issue was larger during the initial stages of the lockdown but tended to decrease with adaptation [78,79]. However, higher-level mental issue were recorded when transitioning to the post-lockdown stage, where individuals were required to integrate the return to a pre-pandemic lifestyle with newly acquired needs (e.g., “new normal”) [80,81,82]. Thus, a part of individuals might suffer from large stress due to transforming their lifestyles among periods, pre-pandemic, restriction during the pandemic and transitioning to new normal after the pandemic.

This study has several limitations. As it is impossible to directly collect suicide motives from victims, the suicide numbers disaggregated by suicidal motives in the SSNPA may be incorrectly estimated due to a potential bias. However, to eliminate subjectivity as much as possible, the judicial police investigate suicide motives based on various pieces of evidence, suicide notes, official documentation (e.g., medical certificates and clinical recordings), and testimony from the victim’s family. Although motive-unidentified suicides were homogeneous among sex/age groups, when the actual motives in motive-unidentified suicides are biased toward specific motives, the results of the analysis may be overestimated or underestimated. Despite these limitations, the SSNPA is considered the most reliable governmental suicide database in Japan, since data were collected by the NPA using consistent investigation methods from 2007 to 2022.

## 5. Conclusions

This study revealed the age/sex-dependent impacts of suicidal motives on SMRP fluctuations of the working-age generation in Japan from 2007 to 2022. Mental health problems were the leading motives with an age-dependent increase in their impact on SMRPs. Economic-related, employment-related, and romance-related problems contributed to the increasing SMRPs of males of 20–29 years in the late 2010s. Romance- and family-related problems contributed to the increasing SMRPs of females of 20–29 in the late 2010s. In particular, despite decreasing birthrates, the increasing SMRPs of females of 20–39 years caused by child-raising stress also continuously increased from the late 2010s. The discrepancy between SMRPs of high-risk groups and males of 30–59 years suggests recent socioeconomic/psychosocial problems contribute to increasing SMRPs in these groups independently of pandemic-associated factors, but the SMRPs of males of 30–59 years were probably affected by factors associated with the ending of the pandemic rather than pandemic-associated factors.

## Data Availability

All raw data are publicly available to any persons via Japanese national databases from national databases of the ‘Suicide Statistics’ (SSNPA) collected by the National Police Agency, the ‘Regional Statistics Database’ of Statistics Bureau of the Ministry of Internal Affairs and Communications (SBMIAC), the ‘Basic Survey on Wage Structure’ of MHLW and the ‘White Paper on Gender Equality 2023′ of Gender Equality Bureau in Cabinet Office.

## Supplementary Materials

The following supporting information can be downloaded at: https://www.mdpi.com/article/10.3390/ejihpe13120193/s1, Supplementary Table S1: SMRPs of 20–29 years disaggregated by motives and sex during 2007–2022; Supplementary Table S2: SMRPs of 30–39 years disaggregated by motives and sex during 2007–2022; Supplementary Table S3: SMRPs of 40–49 years disaggregated by motives and sex during 2007–2022; Supplementary Table S4: SMRPs of 50–59 years disaggregated by motives and sex during 2007–2022.
