# Supplementary material for: Trends in Suicidal Mortality and Motives among Working-Ages Individuals in Japan during 2007–2022
Source: Eur J Investig Health Psychol Educ. 2023 Nov 27;13(12):2795–810. doi: 10.3390/ejihpe13120193 (PMC10742659; doi:10.3390/ejihpe13120193)
Supplement: Supplementary file 1 [file ejihpe-13-00193-s001.zip › ejihpe-2683074-supplementary.pdf]

Supplementary Materials

Supplementary Table S1: SMRPs of 20-29 years disaggregated by motives and sex during 2007-2022 (p2).
Supplementary Table S2: SMRPs of 30-39 years disaggregated by motives and sex during 2007-2022 (p3).
Supplementary Table S3: SMRPs of 40-49 years disaggregated by motives and sex during 2007-2022 (p4).
Supplementary Table S4: SMRPs of 50-59 years disaggregated by motives and sex during 2007-2022 (p5).

Supplementary Table S1: SMRPs of 20-29 years disaggregated by motives and sex during 2007-2022.

	Males		Females	
	count	(min / max)	count	(min / max)
**Total counts of suicide motives during 2007-2022**	30296	(1438 / 2376)	14641	(645 / 1205)
	mean ± SD	(min / max)	mean ± SD	(min / max)
**Population (x1000)**	6,914 ± 373	(6,521 / 7,755)	6,584 ± 384	(6,162 / 7,419)
**Family problems**	2.78 ± 0.46	(2.07 / 3.40)	1.60 ± 0.28	(1.01 / 2.11)
Conflict with parent/child	0.58 ± 0.18	(0.31 / 1.00)	0.39 ± 0.11	(0.16 / 0.61)
Marital conflict	0.71 ± 0.13	(0.47 / 0.91)	0.28 ± 0.08	(0.13 / 0.44)
Conflict with other family members	0.32 ± 0.07	(0.18 / 0.49)	0.20 ± 0.05	(0.11 / 0.28)
Death of family	0.16 ± 0.06	(0.06 / 0.27)	0.12 ± 0.05	(0.05 / 0.24)
Hopeless for family	0.33 ± 0.10	(0.17 / 0.52)	0.16 ± 0.06	(0.06 / 0.27)
Severe verbal reprimand	0.31 ± 0.09	(0.20 / 0.47)	0.08 ± 0.04	(0.03 / 0.14)
Stress of child-raising	0.02 ± 0.02	(0.00 / 0.09)	0.20 ± 0.05	(0.13 / 0.30)
Physical and/or verbal abuse	0.00 ± 0.01	(0.00 / 0.03)	0.00 ± 0.01	(0.00 / 0.03)
Exhaustion from caring for infirm family	0.02 ± 0.01	(0.00 / 0.05)	0.01 ± 0.01	(0.00 / 0.03)
**Health problems**	7.49 ± 1.64	(5.48 / 10.34)	7.00 ± 1.54	(5.05 / 9.38)
Physical illness	0.60 ± 0.16	(0.42 / 0.88)	0.37 ± 0.09	(0.22 / 0.54)
Depression	3.60 ± 1.14	(2.27 / 5.71)	3.91 ± 1.11	(2.40 / 5.69)
Schizophrenia	1.26 ± 0.44	(0.69 / 1.87)	1.04 ± 0.28	(0.66 / 1.50)
Alcoholism	0.05 ± 0.02	(0.01 / 0.09)	0.04 ± 0.03	(0.00 / 0.11)
Drug abuse	0.05 ± 0.04	(0.00 / 0.12)	0.06 ± 0.03	(0.02 / 0.10)
Other mental illness	1.58 ± 0.23	(1.20 / 2.25)	1.41 ± 0.32	(0.95 / 2.17)
Physical disability	0.10 ± 0.04	(0.03 / 0.18)	0.04 ± 0.03	(0.00 / 0.10)
**Economic problems**	5.31 ± 0.72	(4.14 / 6.56)	0.75 ± 0.19	(0.52 / 1.15)
Bankruptcy	0.01 ± 0.01	(0.00 / 0.02)	0.00 ± 0.00	(0.00 / 0.00)
Business struggling	0.12 ± 0.05	(0.02 / 0.24)	0.01 ± 0.01	(0.00 / 0.03)
Unemployment	0.48 ± 0.25	(0.18 / 1.00)	0.08 ± 0.03	(0.02 / 0.13)
Inability to find employment	1.15 ± 0.41	(0.57 / 1.92)	0.24 ± 0.08	(0.11 / 0.42)
Economic hardships	0.90 ± 0.20	(0.39 / 1.23)	0.16 ± 0.07	(0.08 / 0.31)
Overloaded with debt	1.06 ± 0.30	(0.67 / 1.64)	0.08 ± 0.04	(0.02 / 0.14)
Assumption of excessive debt	0.01 ± 0.02	(0.00 / 0.06)	0.00 ± 0.00	(0.00 / 0.01)
Debt (other)	1.00 ± 0.27	(0.77 / 1.86)	0.11 ± 0.05	(0.05 / 0.19)
Harassment by debt-collectors	0.09 ± 0.04	(0.03 / 0.20)	0.01 ± 0.01	(0.00 / 0.05)
Suicide for death benefit	0.01 ± 0.01	(0.00 / 0.05)	0.00 ± 0.00	(0.00 / 0.00)
**Employment problems**	5.19 ± 0.61	(4.16 / 6.12)	1.23 ± 0.31	(0.70 / 1.93)
Failure at work	0.87 ± 0.16	(0.48 / 1.12)	0.18 ± 0.06	(0.07 / 0.32)
Inter-personal relations at work	1.34 ± 0.20	(1.04 / 1.73)	0.45 ± 0.12	(0.25 / 0.66)
Trouble adjusting to changing work environment	0.61 ± 0.16	(0.40 / 1.06)	0.11 ± 0.08	(0.00 / 0.31)
Work-related fatigue	1.43 ± 0.21	(1.10 / 1.76)	0.31 ± 0.11	(0.19 / 0.60)
**Romantic problems**	2.48 ± 0.48	(1.75 / 3.30)	1.98 ± 0.37	(1.31 / 2.64)
Marital problems	0.20 ± 0.07	(0.07 / 0.32)	0.16 ± 0.06	(0.03 / 0.25)
Heartbreak	1.24 ± 0.29	(0.75 / 1.82)	0.71 ± 0.23	(0.42 / 1.27)
Extra-marital affair	0.16 ± 0.05	(0.07 / 0.25)	0.26 ± 0.07	(0.16 / 0.44)
Conflict in relationship	0.71 ± 0.27	(0.00 / 1.20)	0.71 ± 0.24	(0.00 / 1.11)
**School problems**	2.19 ± 0.35	(1.77 / 2.86)	0.57 ± 0.17	(0.27 / 0.91)
Entrance examination problems	0.11 ± 0.05	(0.03 / 0.20)	0.02 ± 0.02	(0.00 / 0.06)
Worrying about future	0.84 ± 0.17	(0.61 / 1.17)	0.21 ± 0.09	(0.01 / 0.41)
Underachievement	0.90 ± 0.15	(0.65 / 1.09)	0.17 ± 0.08	(0.05 / 0.31)
Interpersonal relations with teachers	0.02 ± 0.02	(0.00 / 0.07)	0.01 ± 0.01	(0.00 / 0.04)
Bullying	0.01 ± 0.01	(0.00 / 0.04)	0.01 ± 0.01	(0.00 / 0.02)
Conflict with classmate	0.10 ± 0.05	(0.01 / 0.17)	0.07 ± 0.03	(0.00 / 0.13)
**Others problems**	1.97 ± 0.29	(1.58 / 2.64)	0.73 ± 0.20	(0.50 / 1.12)
Public disclosure of crime	0.26 ± 0.10	(0.01 / 0.43)	0.03 ± 0.02	(0.00 / 0.06)
Crime victim	0.00 ± 0.01	(0.00 / 0.03)	0.02 ± 0.02	(0.00 / 0.06)
Copycat suicide	0.06 ± 0.03	(0.01 / 0.12)	0.08 ± 0.04	(0.03 / 0.16)
Loneliness	0.53 ± 0.12	(0.30 / 0.74)	0.23 ± 0.07	(0.10 / 0.36)
Neighborhood problems	0.02 ± 0.01	(0.00 / 0.04)	0.01 ± 0.01	(0.00 / 0.03)
**Total**	**27.40 ± 3.74**	**(21.76 / 32.79)**	**13.85 ± 2.45**	**(10.15 / 17.95)**

Supplementary Table S2: SMRPs of 30-39 years disaggregated by motives and sex during 2007-2022.

	Males		Females	
	count	(min / max)	count	(min / max)
**Total counts of suicide motives during 2007-2022**	41413	(1790 / 3706)	17680	(718 / 1563)
	mean ± SD	(min / max)	mean ± SD	(min / max)
**Population (x1000)**	8,505 ± 848	(7,197 / 9,663)	8,157 ± 813	(6,870 / 9,254)
**Family problems**	4.03 ± 0.57	(3.33 / 5.04)	2.48 ± 0.47	(1.81 / 3.46)
Conflict with parent/child	0.43 ± 0.10	(0.25 / 0.65)	0.28 ± 0.08	(0.14 / 0.43)
Marital conflict	1.83 ± 0.27	(1.39 / 2.31)	0.70 ± 0.13	(0.40 / 0.90)
Conflict with other family members	0.43 ± 0.08	(0.26 / 0.54)	0.23 ± 0.09	(0.13 / 0.49)
Death of family	0.25 ± 0.05	(0.18 / 0.35)	0.16 ± 0.05	(0.08 / 0.25)
Hopeless for family	0.40 ± 0.09	(0.21 / 0.52)	0.23 ± 0.07	(0.12 / 0.41)
Severe verbal reprimand	0.17 ± 0.04	(0.11 / 0.26)	0.05 ± 0.03	(0.01 / 0.10)
Stress of child-raising	0.07 ± 0.03	(0.02 / 0.17)	0.61 ± 0.14	(0.40 / 0.92)
Physical and/or verbal abuse	0.01 ± 0.01	(0.00 / 0.04)	0.00 ± 0.01	(0.00 / 0.03)
Exhaustion from caring for infirm family	0.06 ± 0.03	(0.03 / 0.14)	0.04 ± 0.04	(0.00 / 0.14)
**Health problems**	9.81 ± 2.02	(6.68 / 13.44)	7.54 ± 1.76	(5.09 / 10.41)
Physical illness	1.02 ± 0.23	(0.73 / 1.49)	0.52 ± 0.14	(0.29 / 0.79)
Depression	5.03 ± 1.33	(3.06 / 7.30)	4.19 ± 1.30	(2.53 / 6.32)
Schizophrenia	1.73 ± 0.41	(1.07 / 2.36)	1.35 ± 0.27	(0.86 / 1.76)
Alcoholism	0.23 ± 0.09	(0.12 / 0.42)	0.12 ± 0.06	(0.01 / 0.21)
Drug abuse	0.08 ± 0.04	(0.01 / 0.14)	0.05 ± 0.02	(0.01 / 0.09)
Other mental illness	1.41 ± 0.20	(1.17 / 1.92)	1.16 ± 0.21	(0.96 / 1.78)
Physical disability	0.11 ± 0.05	(0.06 / 0.22)	0.05 ± 0.03	(0.01 / 0.10)
**Economic problems**	7.33 ± 1.84	(5.42 / 11.04)	0.81 ± 0.24	(0.46 / 1.25)
Bankruptcy	0.03 ± 0.02	(0.00 / 0.08)	0.00 ± 0.00	(0.00 / 0.01)
Business struggling	0.55 ± 0.21	(0.34 / 1.00)	0.03 ± 0.02	(0.01 / 0.07)
Unemployment	0.90 ± 0.49	(0.38 / 2.11)	0.10 ± 0.06	(0.05 / 0.30)
Inability to find employment	0.58 ± 0.15	(0.40 / 0.85)	0.08 ± 0.03	(0.01 / 0.14)
Economic hardships	1.44 ± 0.20	(1.13 / 1.79)	0.24 ± 0.11	(0.01 / 0.42)
Overloaded with debt	1.73 ± 0.62	(1.08 / 3.29)	0.11 ± 0.07	(0.02 / 0.25)
Assumption of excessive debt	0.04 ± 0.04	(0.00 / 0.13)	0.01 ± 0.01	(0.00 / 0.03)
Debt (other)	1.41 ± 0.46	(0.89 / 2.29)	0.14 ± 0.06	(0.06 / 0.26)
Harassment by debt-collectors	0.16 ± 0.06	(0.06 / 0.27)	0.01 ± 0.01	(0.00 / 0.03)
Suicide for death benefit	0.05 ± 0.03	(0.00 / 0.12)	0.01 ± 0.01	(0.00 / 0.03)
**Employment problems**	5.29 ± 0.68	(4.23 / 6.51)	0.81 ± 0.19	(0.57 / 1.15)
Failure at work	0.88 ± 0.14	(0.59 / 1.08)	0.08 ± 0.05	(0.01 / 0.16)
Inter-personal relations at work	1.36 ± 0.24	(1.00 / 1.97)	0.32 ± 0.08	(0.23 / 0.52)
Trouble adjusting to changing work environment	0.69 ± 0.12	(0.52 / 1.03)	0.09 ± 0.05	(0.04 / 0.22)
Work-related fatigue	1.49 ± 0.20	(1.11 / 1.77)	0.21 ± 0.07	(0.11 / 0.35)
**Romantic problems**	1.96 ± 0.30	(1.38 / 2.54)	1.22 ± 0.32	(0.70 / 1.74)
Marital problems	0.19 ± 0.09	(0.07 / 0.33)	0.14 ± 0.05	(0.05 / 0.23)
Heartbreak	0.69 ± 0.07	(0.51 / 0.79)	0.32 ± 0.11	(0.15 / 0.58)
Extra-marital affair	0.40 ± 0.09	(0.24 / 0.63)	0.29 ± 0.08	(0.13 / 0.40)
Conflict in relationship	0.52 ± 0.17	(0.00 / 0.71)	0.37 ± 0.14	(0.04 / 0.55)
**School problems**	0.06 ± 0.02	(0.02 / 0.11)	0.02 ± 0.02	(0.00 / 0.06)
Entrance examination problems	0.01 ± 0.01	(0.00 / 0.03)	0.00 ± 0.00	(0.00 / 0.01)
Worrying about future	0.02 ± 0.02	(0.00 / 0.06)	0.00 ± 0.00	(0.00 / 0.01)
Underachievement	0.02 ± 0.02	(0.00 / 0.06)	0.01 ± 0.01	(0.00 / 0.02)
Interpersonal relations with teachers	0.00 ± 0.00	(0.00 / 0.01)	0.00 ± 0.00	(0.00 / 0.01)
Bullying	0.00 ± 0.00	(0.00 / 0.01)	0.00 ± 0.00	(0.00 / 0.01)
Conflict with classmate	0.00 ± 0.01	(0.00 / 0.03)	0.00 ± 0.01	(0.00 / 0.01)
**Others problems**	1.65 ± 0.22	(1.25 / 1.98)	0.51 ± 0.09	(0.39 / 0.68)
Public disclosure of crime	0.40 ± 0.08	(0.25 / 0.58)	0.04 ± 0.02	(0.00 / 0.08)
Crime victim	0.01 ± 0.01	(0.00 / 0.03)	0.01 ± 0.01	(0.00 / 0.06)
Copycat suicide	0.06 ± 0.03	(0.01 / 0.11)	0.06 ± 0.02	(0.02 / 0.09)
Loneliness	0.46 ± 0.11	(0.23 / 0.61)	0.14 ± 0.05	(0.04 / 0.25)
Neighborhood problems	0.02 ± 0.02	(0.00 / 0.05)	0.03 ± 0.02	(0.00 / 0.06)
**Total**	**30.12 ± 4.96**	**(24.21 / 38.94)**	**13.38 ± 2.69**	**(9.66 / 17.39)**

Supplementary Table S3: SMRPs of 40-49 years disaggregated by motives and sex during 2007-2022.

	Males				Females			
	count		min	max	count		min	max
**Total counts of suicide motives during 2007-2022**	53749		2442	4374	19934		1020	1529
	mean	SD	min	max	mean	SD	min	max
**Population (x1000)**	9,035	± 630	7,907	9,784	8,776	± 604	7,715	9,506
**Family problems**	5.16	± 0.99	3.77	7.03	2.74	± 0.49	2.12	3.97
Conflict with parent/child	0.45	± 0.10	0.29	0.66	0.36	± 0.08	0.22	0.51
Marital conflict	2.20	± 0.44	1.62	3.11	0.72	± 0.18	0.42	1.06
Conflict with other family members	0.57	± 0.13	0.38	0.78	0.25	± 0.05	0.14	0.36
Death of family	0.42	± 0.09	0.30	0.60	0.28	± 0.07	0.18	0.45
Hopeless for family	0.63	± 0.11	0.46	0.81	0.36	± 0.07	0.22	0.53
Severe verbal reprimand	0.14	± 0.04	0.08	0.26	0.05	± 0.02	0.02	0.09
Stress of child-raising	0.10	± 0.06	0.01	0.24	0.37	± 0.12	0.18	0.70
Physical and/or verbal abuse	0.00	± 0.01	0.00	0.02	0.00	± 0.01	0.00	0.03
Exhaustion from caring for infirm family	0.18	± 0.09	0.01	0.33	0.13	± 0.06	0.01	0.25
**Health problems**	12.31	± 2.49	8.83	16.62	8.54	± 1.39	6.45	10.71
Physical illness	2.01	± 0.62	1.21	3.02	0.98	± 0.21	0.66	1.29
Depression	6.34	± 1.58	4.24	9.14	4.72	± 1.08	3.20	6.40
Schizophrenia	1.64	± 0.28	1.07	2.00	1.40	± 0.16	1.15	1.69
Alcoholism	0.46	± 0.17	0.21	0.77	0.15	± 0.05	0.01	0.23
Drug abuse	0.08	± 0.04	0.01	0.18	0.04	± 0.02	0.01	0.08
Other mental illness	1.33	± 0.25	0.93	2.15	1.07	± 0.21	0.76	1.64
Physical disability	0.21	± 0.06	0.11	0.37	0.05	± 0.03	0.01	0.10
**Economic problems**	11.34	± 5.02	6.24	20.64	1.21	± 0.40	0.74	1.93
Bankruptcy	0.11	± 0.09	0.02	0.38	0.01	± 0.01	0.00	0.03
Business struggling	1.44	± 0.76	0.67	2.79	0.07	± 0.04	0.01	0.14
Unemployment	1.32	± 0.86	0.43	3.08	0.11	± 0.07	0.03	0.26
Inability to find employment	0.43	± 0.15	0.23	0.75	0.06	± 0.04	0.00	0.14
Economic hardships	2.34	± 0.62	1.59	3.55	0.42	± 0.10	0.25	0.60
Overloaded with debt	2.58	± 1.50	1.35	5.83	0.18	± 0.13	0.07	0.55
Assumption of excessive debt	0.07	± 0.07	0.00	0.21	0.01	± 0.01	0.00	0.05
Debt (other)	2.18	± 1.05	1.08	4.07	0.21	± 0.12	0.05	0.44
Harassment by debt-collectors	0.20	± 0.14	0.07	0.57	0.02	± 0.02	0.00	0.06
Suicide for death benefit	0.19	± 0.14	0.03	0.46	0.02	± 0.01	0.00	0.05
**Employment problems**	5.94	± 1.08	4.38	7.76	0.68	± 0.18	0.45	1.20
Failure at work	1.00	± 0.23	0.67	1.40	0.07	± 0.04	0.02	0.15
Inter-personal relations at work	1.34	± 0.26	0.97	1.95	0.25	± 0.07	0.12	0.43
Trouble adjusting to changing work environment	0.92	± 0.29	0.58	1.81	0.10	± 0.06	0.05	0.29
Work-related fatigue	1.74	± 0.28	1.24	2.23	0.14	± 0.06	0.01	0.24
**Romantic problems**	1.22	± 0.22	0.79	1.52	0.65	± 0.16	0.48	1.01
Marital problems	0.11	± 0.04	0.05	0.20	0.06	± 0.04	0.01	0.14
Heartbreak	0.30	± 0.09	0.18	0.51	0.14	± 0.04	0.08	0.25
Extra-marital affair	0.32	± 0.08	0.14	0.44	0.18	± 0.06	0.08	0.28
Conflict in relationship	0.35	± 0.13	0.00	0.50	0.20	± 0.08	0.01	0.34
**School problems**	0.01	± 0.01	0.00	0.03	0.01	± 0.01	0.00	0.05
Entrance examination problems	0.00	± 0.00	0.00	0.01	0.00	± 0.00	0.00	0.01
Worrying about future	0.00	± 0.00	0.00	0.01	0.00	± 0.01	0.00	0.02
Underachievement	0.00	± 0.00	0.00	0.01	0.00	± 0.00	0.00	0.01
Interpersonal relations with teachers	0.00	± 0.00	0.00	0.00	0.00	± 0.00	0.00	0.01
Bullying	0.00	± 0.00	0.00	0.01	0.00	± 0.00	0.00	0.01
Conflict with classmate	0.00	± 0.00	0.00	0.00	0.00	± 0.00	0.00	0.00
**Others problems**	1.70	± 0.27	1.25	2.17	0.49	± 0.14	0.19	0.73
Public disclosure of crime	0.47	± 0.10	0.33	0.64	0.05	± 0.02	0.02	0.10
Crime victim	0.01	± 0.01	0.00	0.03	0.01	± 0.01	0.00	0.03
Copycat suicide	0.07	± 0.03	0.01	0.15	0.06	± 0.04	0.00	0.14
Loneliness	0.47	± 0.17	0.04	0.68	0.16	± 0.06	0.06	0.25
Neighborhood problems	0.04	± 0.02	0.02	0.07	0.03	± 0.02	0.00	0.07
**Total**	**37.66**	**± 9.51**	**25.63**	**53.29**	**14.32**	**± 2.40**	**10.92**	**18.42**

Supplementary Table S4: SMRPs of 50-59 years disaggregated by motives and sex during 2007-2022.

	Males				Females			
	count		min	max	count		min	max
**Total counts of suicide motives during 2007-2022**	58511		2431	5605	20813		1046	1685
	mean	SD	min	max	mean	SD	min	max
**Population (x1000)**	8,214	± 495	7,753	9,420	8,183	± 503	7,712	9,447
**Family problems**	5.21	± 1.01	3.55	6.83	2.88	± 0.57	2.31	4.47
Conflict with parent/child	0.45	± 0.12	0.31	0.81	0.35	± 0.08	0.25	0.57
Marital conflict	1.71	± 0.31	1.29	2.34	0.57	± 0.12	0.43	0.90
Conflict with other family members	0.61	± 0.20	0.33	0.91	0.31	± 0.08	0.20	0.46
Death of family	0.64	± 0.10	0.45	0.89	0.42	± 0.09	0.23	0.64
Hopeless for family	0.75	± 0.23	0.35	1.13	0.50	± 0.12	0.30	0.68
Severe verbal reprimand	0.12	± 0.05	0.05	0.22	0.05	± 0.02	0.02	0.09
Stress of child-raising	0.08	± 0.04	0.00	0.15	0.11	± 0.07	0.03	0.34
Physical and/or verbal abuse	0.01	± 0.01	0.00	0.05	0.00	± 0.01	0.00	0.01
Exhaustion from caring for infirm family	0.44	± 0.11	0.25	0.69	0.32	± 0.08	0.16	0.48
**Health problems**	15.45	± 3.28	11.07	20.96	9.99	± 1.16	8.03	11.78
Physical illness	4.65	± 1.94	2.28	8.05	1.85	± 0.33	1.45	2.41
Depression	6.83	± 1.14	4.99	8.82	5.51	± 0.84	4.21	6.94
Schizophrenia	1.30	± 0.18	1.02	1.63	1.25	± 0.19	0.88	1.60
Alcoholism	0.65	± 0.19	0.39	1.02	0.12	± 0.05	0.02	0.21
Drug abuse	0.05	± 0.02	0.00	0.08	0.03	± 0.02	0.00	0.06
Other mental illness	1.30	± 0.27	1.02	2.06	1.00	± 0.31	0.67	1.93
Physical disability	0.38	± 0.13	0.22	0.67	0.11	± 0.05	0.02	0.21
**Economic problems**	15.81	± 7.18	8.23	29.67	1.60	± 0.46	1.10	2.58
Bankruptcy	0.18	± 0.12	0.01	0.38	0.01	± 0.01	0.00	0.03
Business struggling	2.50	± 1.47	0.87	5.09	0.16	± 0.08	0.07	0.32
Unemployment	1.78	± 1.02	0.79	3.92	0.10	± 0.04	0.04	0.17
Inability to find employment	0.43	± 0.19	0.24	0.87	0.03	± 0.03	0.00	0.08
Economic hardships	3.69	± 1.06	2.50	6.34	0.56	± 0.12	0.41	0.92
Overloaded with debt	3.14	± 1.71	1.37	6.42	0.22	± 0.16	0.01	0.56
Assumption of excessive debt	0.09	± 0.09	0.01	0.29	0.01	± 0.02	0.00	0.05
Debt (other)	2.80	± 1.42	1.19	5.58	0.29	± 0.15	0.13	0.61
Harassment by debt-collectors	0.20	± 0.13	0.01	0.51	0.02	± 0.02	0.00	0.06
Suicide for death benefit	0.32	± 0.21	0.11	0.67	0.03	± 0.02	0.00	0.08
**Employment problems**	5.55	± 0.84	4.16	7.50	0.54	± 0.20	0.26	1.09
Failure at work	1.06	± 0.21	0.74	1.38	0.06	± 0.02	0.02	0.11
Inter-personal relations at work	1.03	± 0.25	0.67	1.80	0.19	± 0.09	0.10	0.40
Trouble adjusting to changing work environment	0.89	± 0.22	0.64	1.55	0.08	± 0.06	0.01	0.27
Work-related fatigue	1.69	± 0.24	1.08	1.96	0.12	± 0.06	0.01	0.21
**Romantic problems**	0.63	± 0.11	0.46	0.84	0.27	± 0.07	0.18	0.41
Marital problems	0.05	± 0.02	0.01	0.09	0.02	± 0.02	0.00	0.06
Heartbreak	0.11	± 0.04	0.06	0.20	0.04	± 0.02	0.01	0.08
Extra-marital affair	0.18	± 0.08	0.01	0.33	0.10	± 0.03	0.06	0.17
Conflict in relationship	0.19	± 0.07	0.00	0.28	0.07	± 0.04	0.00	0.15
**School problems**	0.00	± 0.00	0.00	0.01	0.00	± 0.00	0.00	0.01
Entrance examination problems	0.00	± 0.00	0.00	0.00	0.00	± 0.00	0.00	0.00
Worrying about future	0.00	± 0.00	0.00	0.00	0.00	± 0.00	0.00	0.00
Underachievement	0.00	± 0.00	0.00	0.00	0.00	± 0.00	0.00	0.00
Interpersonal relations with teachers	0.00	± 0.00	0.00	0.00	0.00	± 0.00	0.00	0.00
Bullying	0.00	± 0.00	0.00	0.00	0.00	± 0.00	0.00	0.00
Conflict with classmate	0.00	± 0.00	0.00	0.00	0.00	± 0.00	0.00	0.00
**Others problems**	1.85	± 0.39	1.31	2.48	0.57	± 0.18	0.38	1.12
Public disclosure of crime	0.41	± 0.06	0.33	0.51	0.05	± 0.03	0.00	0.09
Crime victim	0.01	± 0.01	0.00	0.04	0.00	± 0.01	0.00	0.01
Copycat suicide	0.10	± 0.04	0.01	0.18	0.07	± 0.03	0.03	0.11
Loneliness	0.62	± 0.19	0.35	1.06	0.24	± 0.09	0.09	0.46
Neighborhood problems	0.08	± 0.04	0.03	0.16	0.03	± 0.02	0.00	0.10
**Total**	**44.51**	**± 12.07**	**29.42**	**65.16**	**15.86**	**± 1.99**	**13.02**	**19.68**
